# Quantifying Topological Uncertainty in Fractured Systems using Graph Theory and Machine Learning

**DOI:** 10.1038/s41598-018-30117-1

**Published:** 2018-08-03

**Authors:** Gowri Srinivasan, Jeffrey D. Hyman, David A. Osthus, Bryan A. Moore, Daniel O’Malley, Satish Karra, Esteban Rougier, Aric A. Hagberg, Abigail Hunter, Hari S. Viswanathan

**Affiliations:** 1 Theoretical Division, Los Alamos National Laboratory, Los Alamos, NM 87545 USA; 2 Earth and Environmental Sciences Division, Los Alamos National Laboratory, Los Alamos, NM 87545 USA; 3 Computer, Computational and Statistical Sciences Division, Los Alamos National Laboratory, Los Alamos, NM 87545 USA; 4 X-Computational Physics Division, Los Alamos National Laboratory, Los Alamos, NM 87545 USA

## Abstract

Fractured systems are ubiquitous in natural and engineered applications as diverse as hydraulic fracturing, underground nuclear test detection, corrosive damage in materials and brittle failure of metals and ceramics. Microstructural information (fracture size, orientation, etc.) plays a key role in governing the dominant physics for these systems but can only be known statistically. Current models either ignore or idealize microscale information at these larger scales because we lack a framework that efficiently utilizes it in its entirety to predict macroscale behavior in brittle materials. We propose a method that integrates computational physics, machine learning and graph theory to make a paradigm shift from computationally intensive high-fidelity models to coarse-scale graphs without loss of critical structural information. We exploit the underlying discrete structure of fracture networks in systems considering flow through fractures and fracture propagation. We demonstrate that compact graph representations require significantly fewer degrees of freedom (dof) to capture micro-fracture information and further accelerate these models with Machine Learning. Our method has been shown to improve accuracy of predictions with up to four orders of magnitude speedup.

## Introduction

Fractures are a foundational structure in numerous natural and engineered applications that influence our daily lives. Examples that motivated this study include (1) hydraulic fracturing, which has had a profound impact on US energy independence through the increased availability of unconventional fossil fuels^1,2^; (2) chemical signature from clandestine nuclear weapon testing, where gas migration through fractured rock provides the definitive smoking gun when used in conjunction with conventional seismic methods^3^ and remains critical to global security as countries like North Korea continue to conduct low-yield nuclear tests; and (3) predicting the brittle failure of materials such as ceramics and some metals, e.g., corrosive damage in materials and brittle failure of ceramics in airplane wings, spacecraft tiles.

For all of the examples of fractured systems mentioned here, individual fracture information (geometry, orientation etc.), despite being critical to macroscale behavior^4^, can only be known in a statistical sense. In fractured systems, the connections between fractures often dominate system behavior, we refer to this connectivity as the topology of the fracture network. Because the fracture networks are statistically modeled, the topology is inherently uncertain and requires an ensemble of realizations of these fracture networks. Since the topology of the graph matches the topology of the fracture network, we can interrogate the topological uncertainty with the graph. Moreover, the uncertainty surrounding topological properties (network connectivity) dominate system behavior. For example, in a system with two large fractures, system behavior is very much dependent on whether these fractures intersect or are connected by smaller fractures. However, such structural information cannot be fully characterized at the macroscale due to the high computational cost incurred in representing the discontinuities formed by the presence of cracks using highly resolved meshes. While there are high fidelity mesh-based fracture models capable of representing millions of micro-fractures, such as dfnWorks^5^ and HOSS^6^ (both developed by this team), the computational cost for 1000s of model runs to bound the topological uncertainty quickly adds up to petabytes of information and is not feasible^7,8^. Therefore, many researchers have turned to reduced order models (ROM) to represent these systems, but a general framework linking attributes in the high-fidelity models to the induced ROM is still lacking.

We present a general methodology here to account for the critical aspect of network structure in ROMs in fractured geo-materials using a hybrid graph theoretical/machine learning (ML) approach. The novel research contribution highlighted here is the general framework we have developed, demonstrated on two separate applications in brittle geomaterials, where the common theme is the importance of the underlying fracture network structure in governing the dominant physics. Although the applications mentioned here appear to be very different problems occurring at different scales, the technical challenge is similar-representing the relevant physics on the graph through ML algorithms and quantifying the dominant topological uncertainty. Graph theory is a powerful tool for interrogating structured systems. Across many disciplines, ML has proven to simplify and expedite previously computationally intensive processes by learning from available data and knowledge. Combined with graph theory, ML approaches can effectively tackle a broad set of problems in fractured systems where quantifying uncertainties due to the topology is critical.

In this study we bridge the knowledge gap between the discrete (micron-mm) and continuum (cm-m) scales efficiently by exploiting the underlying structure of fracture networks. We formulate compact graph representations of fracture networks that avoid detailed meshing and require 2–3 orders of magnitude fewer degrees of freedom (dof) to capture micro-fracture information. Recent work in network theory has shown its utility for problems such as diffusion and percolation^9^ as well as failure problems^10^, which are similar to topics we explore. By combing ML with graph theory, we develop an approach that efficiently tackles a broad set of problems in fractured systems where structure and topology are critical. Our method seamlessly lends itself to an uncertainty quantification (UQ) framework that requires a fraction of the computational resources.

In order to demonstrate the robustness and utility of the method, we apply it to two important geophysical problems, flow through fractured media and fracture propagation. Our critical advance is to integrate computational physics, machine learning and graph theory to make a paradigm shift from computationally intensive grid-based models to efficient graphs. Our graph-based algorithms have made it possible to directly extract geophysical and topological features used in the ML algorithms to predict key phenomena that drive the underlying physics. We investigate several topological metrics using graph representations and identify those that are appropriate for the different applications we consider. The graph-based algorithms make it possible to extract geophysical and topological features for use in the ML algorithms to predict key phenomena that drive the underlying physics. Our key finding is that appropriately configured graph-based reduced order models can maintain the accuracy of the high-fidelity models with up to 4 orders of magnitude speedup in computational cost. We also harness the power of ML algorithms to reveal previously neglected, but key microstructural effects and derive accurate upscaled parameters for use in continuum models. For example, continuum-scale material models often only consider one dominant crack orientation, or just one crack and no interactions. The proposed hybrid graph-theoretical/machine learning approach captures these interactions, which are critical in high-fidelity discrete simulations and allow the extracted information to be incorporated into continuum scale models. We demonstrate that combining ML and graph-based approaches makes such a framework possible.

## Results

Our approach is based on verifying the following hypotheses: (1) Primary flow paths can be identified a priori with graph-based methods, confining computational power to critical regions of interest; (2) Predictive uncertainty is dominated by the topology as a result of structural effects; and (3) Dominant emergent phenomena related to fracture interaction and coalescence can be predicted using ML methods that use feature importance identification mechanisms since the geometry and topology of the fracture networks are directly represented in the graphs as features. We demonstrate our advances in proving these hypotheses in the next three sub-sections.

### Ascertaining the Topological Characteristics of a Fracture Network using graph-based physics solutions and ML-based pruning

We first address the hypothesis regarding the pruning of a fracture network to only include the regions that participate significantly in the governing physics. We take on the challenging task of identifying primary flow paths through a fracture network a priori, without conducting computationally intensive mesh-based computations. High-fidelity simulations can then be used efficiently to focus on the primary flow path without including the extraneous parts of the domain where little or no flow occurs. Here our Quantity of Interest (QOI) is the first passage time of a solute being transported along with the flow field. For the exposition of our methods, we adopt a Lagrangian setting where the solute plume is represented by a cloud of tracer particles and the breakthrough curve (BTC) is the cumulative density function of the time it takes for a particle to travel from the inlet boundary to the outlet boundary. Field and laboratory experiments of flow through fracture networks indicate that flow channeling is a common feature through fractured subsurface systems^11^ strongly suggesting the existence of primary flow pathways. Casting the discrete fracture network (DFN) as a graph representation allows us to identify relevant sub-networks of the entire network based solely on topology, and here we present three ways to prune the domain – specifically 2-core, shortest paths, and an ML classification approach. Representing the fracture network as a graph allows us to use existing graph theoretic algorithms while introducing a rich feature set that can be leveraged by ML algorithms. In this graph-representation, fractures in the DFN are represented as nodes in the graph and if two fractures intersect then there is an edge in the graph connecting the corresponding nodes^7^.

**Figure 1:**
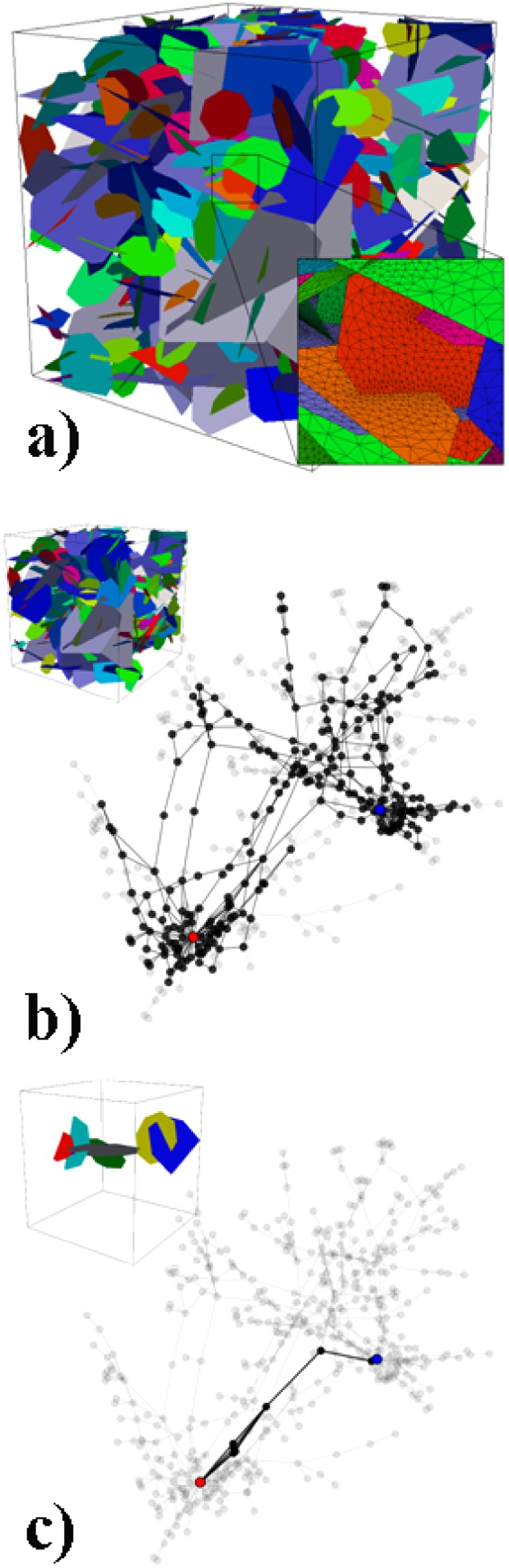
A modest sized fracture network with 459 fractures. (**a**) The original Discrete Fracture Network (DFN) model; (**b**) the 2-core representation of the DFN and (**c**) the graph corresponding to the shortest path between inflow and outflow boundaries. Insets show the DFN models corresponding to the reduced graph representations.

Figure 1a shows a modest sized synthetically generated DFN made up of 459 fractures whose lengths are sampled from a power-law distribution, representative of real world fracture networks^12^, with centroids and orientations drawn from uniform random distributions. Fracture apertures vary between fractures and are positively correlated to the fracture radius (a common assumption in DFN modeling supported by field observations^13–15^). The inset in Fig. 1a, shows the mesh and in particular, illustrates the acute refinement at fracture intersections needed to accurately resolve the high-pressure gradients that occur in these regions. The first pruning algorithm isolates the 2-core of the graph, which is the maximal subgraph such that every node has degree 2 or more^16^, as a relevant part of the domain that participates in the flow, shown in Fig. 1b. Source and target nodes that represent the inflow and outflow boundaries are shown in red and blue respectively and connect to nodes that represent fractures which intersect those boundaries. The graph full network is shown semi-transparent for reference. The 2-core of this DFN is made up of 254 fractures, which is a reduction of 45% in number of fractures. An alternate way to prune the domain is by retaining only the shortest path in the network from the source to the target, which is shown along with the equivalent graph in Fig. 1c. In this case, the resulting shortest path network is made up of only 7 fractures.

**Figure 2:**
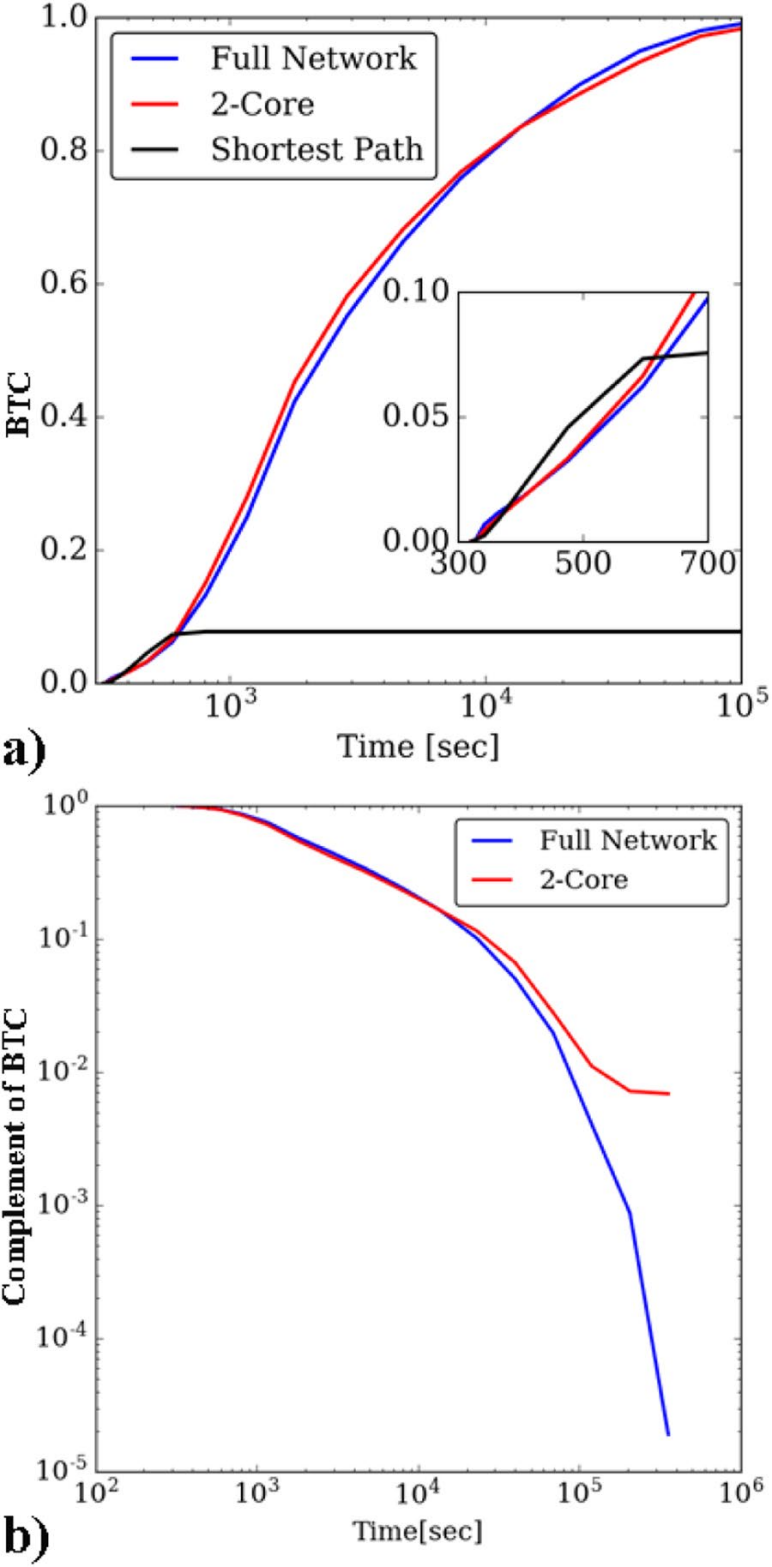
(**a**) Comparison of BTCs from the full DFN network, 2-core, and shortest path graph representations and (**b**) Complement of the BTC shows difference in tailing behavior between full DFN and 2-Core representation.

In order to test how well the sub-networks represent the original DFN, we perform a comparison of upscaled properties. The BTC computed under the same boundary conditions for flow through the full (blue) and the 2-core (red), and shortest path (black) fracture networks are plotted together in Fig. 2 as a function of time. Despite having 45% fewer fractures than the original network, the breakthrough curve of particles passing through the 2-core of the network closely resembles that of the full network. This similarity, which can be observed by plotting the complement of the BTC (Fig. 2b), persists except at very late times. This is consistent with discarding trees in the graph that cause dispersion into and out of dead ends leading to late arrivals.

Transport through the shortest path (black line Fig. 2a) network is a strong indicator for the earliest breakthrough times of the full network; the first particle breakthrough of the shortest path is within 2% of that obtained in the full network. These results show accurate graph-based models are capable of identifying primary flow paths and hence an appropriate reduced domain based on the application of interest. We explored how increasing the number of shortest paths retained influenced the accuracy of predicting the first breakthrough times^7^. We demonstrated how to incorporate network properties into this selection for more robust predictions. We also performed ML on our graph-based models to better identify the sub-network that corresponds to fractures along the primary flowing paths^17^. We used supervised classification methods, specifically support vector machine and random forest algorithms, to identify the flowing backbones from our DFN models. In contrast to DFN models that can take 10 s of hours per realization, these ML methods require only minutes to train and, after training, require merely seconds to identify the flowing backbone. Using these classification methods we obtained pruned networks with around 25% of the fractures in the original network demonstrating that combining ML and graphs results in a powerful tool for emulating high-fidelity simulations of structured systems with a vastly decreased computational cost. The ML approach provides more pruning than the 2-core method while retaining accuracy. These results make major strides towards proving our first hypothesis: primary flow paths can be identified a priori with graph-based methods, confining computational power to critical regions of interest.

### Quantification of Topological Uncertainty

In an effort to further reduce computational burden, we exploit graph-based reduced order models as an appealing mesh-free alternative, where flow and transport calculations are performed on the equivalent graph representation. Our recently developed graph Laplacian solver can simulate transport of conservative solutes through a fracture network^18^ by mapping intersections to nodes and fracture segments to edges, and up to 4 orders of magnitude computational speedup is achieved with accuracy tradeoffs. These graph representations include in-fracture attributes, e.g., lengths between intersections and fracture apertures, as edge-weights. Deviations in transport properties on the graph from the high-fidelity model are systematic. We take advantage of the systematic nature of the deviations by using a Bayesian UQ methodology^19^ that quantifies system uncertainties represented by the deviations in the BTCs even when our computationally efficient graph-based reduced order models are not an exact representation of the high-fidelity model. Furthermore, and nontrivially, our Bayesian calibration approach accurately quantifies the uncertainty in the predictions of calibrated QOIs.

We demonstrate our approach on an ensemble of 100 high-fidelity DFN simulations, generated in the same manner as the network in Fig. 1. We refer to the high fidelity DFN as F, and the graphical representation of the DFN as G. We relate high-fidelity DFN BTC_F to its graph-based counterpart BTC_G, via calibration parameters and a discrepancy^20^. Finally, given a BTC_G, we modify it through calibration and a discrepancy adjustment, resulting in a prediction for BTC_F with uncertainty. We use a subset of the 100 networks to learn the discrepancy and calibration terms and the rest for testing the quality of predictions.

**Figure 3:**
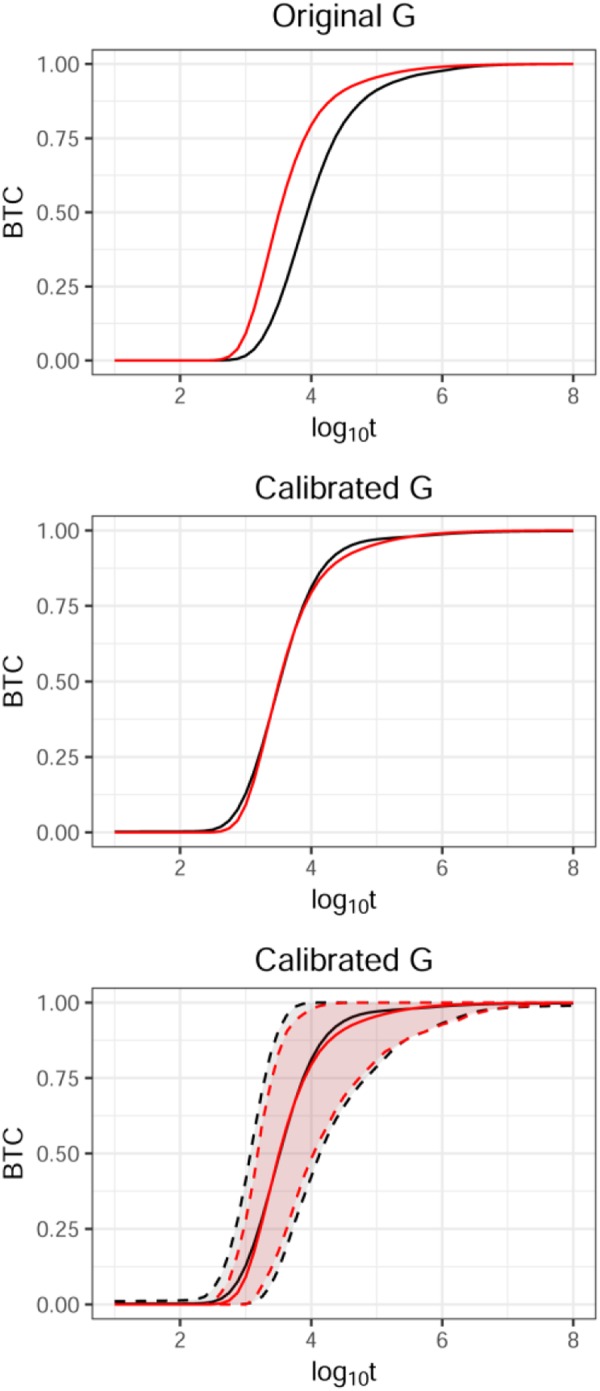
Calibration process for reduced graph-based BTC (Top) Discrepancy between graph-based BTC_G in black and DFN-based BTC_F in red (Middle) BTC_G calibrated to match BTC_F (Bottom) Predictions of BTC_F based on calibrated BTC_G with uncertainties closely match ensemble statistics of directly computed DFN-based BTC.

Figure 3a shows the deviation of the mean BTC of the ensemble obtained from our graph-based transport solver from the ensemble BTC generated using the dfnWorks suite^5^. Our Bayesian methodology corrects the deviation using a single calibration parameter, learned with uncertainty, to shift the BTC in time, and adds a discrepancy function to minimize any deviations thereafter. The resulting mean BTC is shown in Fig. 3b. Finally Fig. 3c shows the statistics of the system, represented by the ensemble of fracture networks generated in this study. The system uncertainty, characterized by a mean and the 95% prediction interval, which would typically be bounded by simulating a hundred DFNs, is shown in red. The corresponding ensemble uncertainty predicted using the corrected graph-based BTCs is shown in black. The close match between the statistics of the system represented by F and G indicate that very few BTC_F/BTC_G pairs are needed to correct for the discrepancies and bound overall system uncertainties using the reduced order models. These results demonstrate our second hypothesis that predictive uncertainty is dominated by structural effects but spans topological uncertainty space.

### Dynamic Fracture Propagation

**Figure 4:**
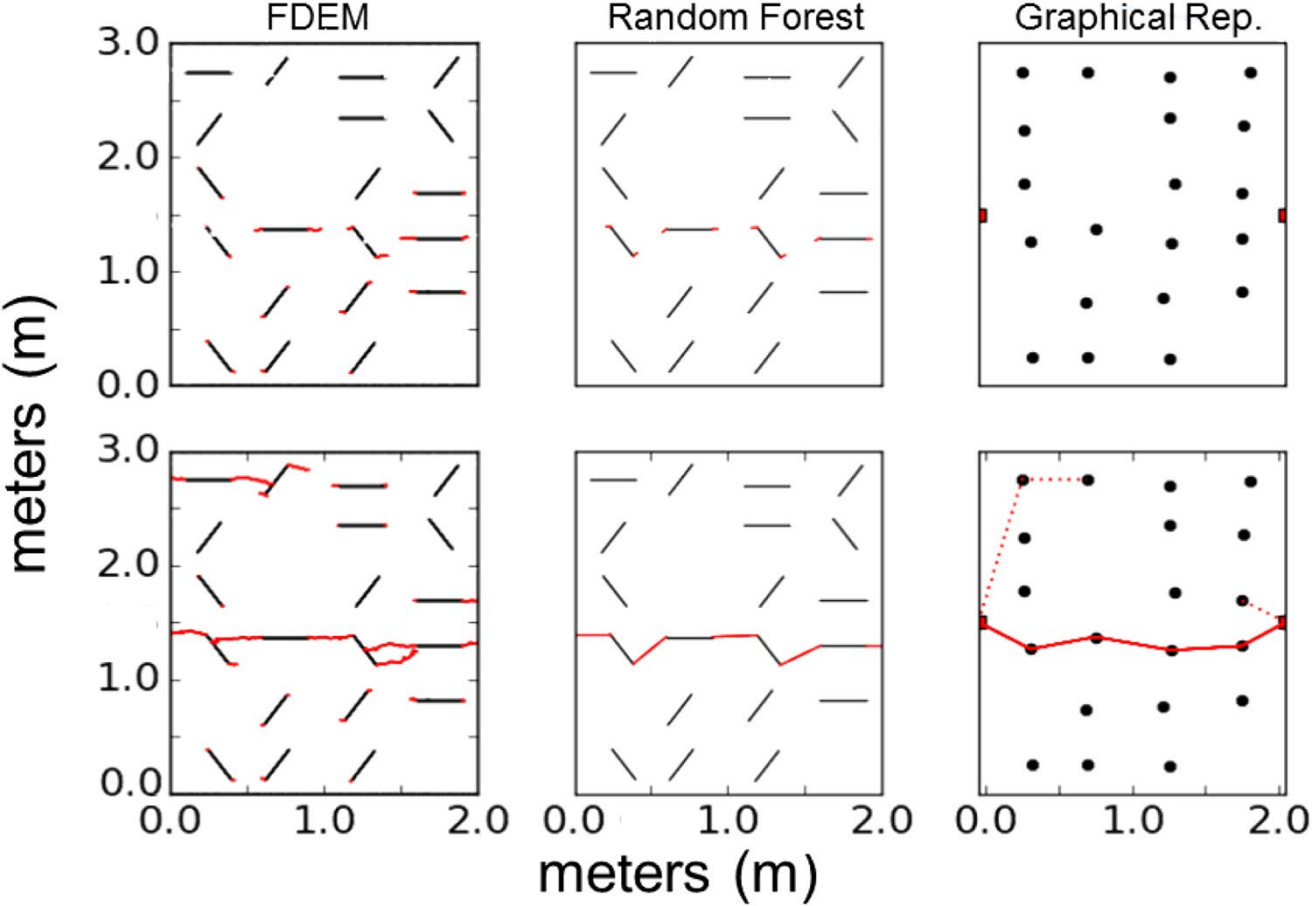
Comparison of HOSS simulation to ML predictions and the graphical representation are shown here at (top) an early time and (bottom) failure. The solid line lines in the bottom right panel represents the path to failure which is accurately predicted by the Random Forest model. The dashed indicates crack growth and coalescence in the HOSS simulations which are not captured in the Random Forest model.

Next, we exploit the nascent field of dynamic graphs combined with ML to develop reduced order models for the more complex case where fractures evolve with time^8^. Currently, reduced order formulations, which include semi-analytical models and continuum approximations, do not account for crack interactions leading to significant errors in failure predictions, particularly resulting in non-conservative predictions. Times to failure are typically over-predicted resulting in failure before it is expected. Here, we define time to failure to be the amount of time that elapses between when the loading process begins and when a connected fracture spans the entire sample, e.g., in the lower left of Fig. 4. The eventual goal of these simulations is to predict the evolution of the effective moduli of the material as cracks grow and coalesce leading to failure of the material.

The first step in formulating a more accurate material model is learning how crack interactions influence the time to failure, and determining characteristics of preferential paths to failure. We generate crack growth and interaction data from running several simulations of HOSS, a computationally expensive, high-fidelity crack evolution model that can resolve individual micro-cracks unlike the macro-scale continuum models. HOSS accounts for interactions between micro-cracks in addition to coalescence and growth damage evolution mechanisms. We identify key features in the crack growth data (orientation, geometry, etc.) and map the evolving crack network into a dynamic graph model, where cracks are represented by a node and edges correspond to intersecting cracks. The data is partitioned for training and validation purposes and tested on simple fracture systems. The ML algorithms employed were Decision Trees (DT) and Random Forests (RF) and samples were seeded with 20 initial micro-cracks. These algorithms provide great insight on feature importance within the model and data^21,22^. These predictive tools were compared with the high fidelity results (HOSS results).

**Figure 5:**
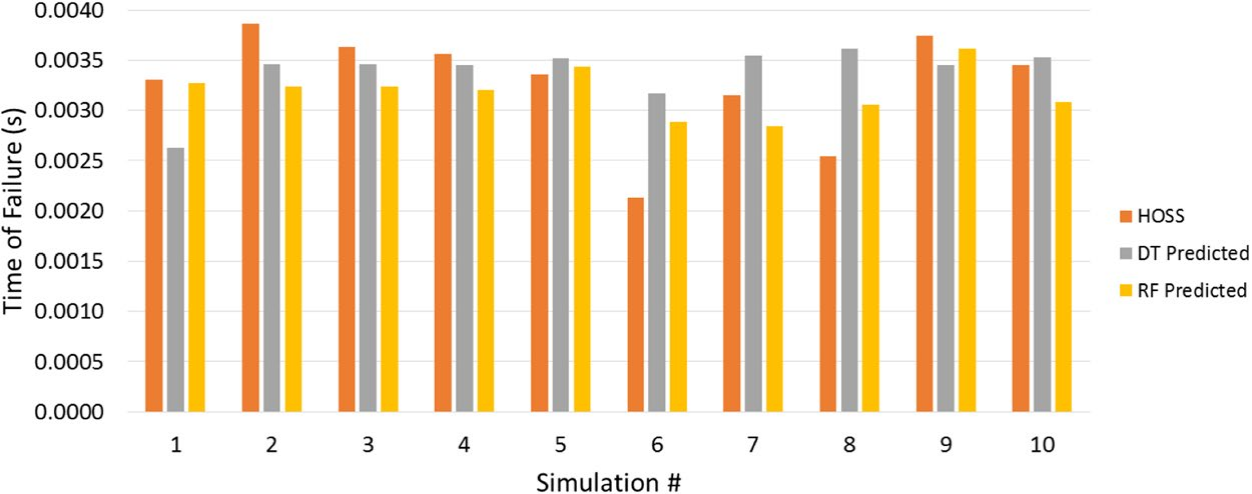
Comparison of high fidelity model HOSS to graph-based ML reduced order models using Decision Trees (DT) and Random Forest (RF).

In the test cases studied, the sample size was 2 m × 3 m, with tensile loading at the top boundary, holding the bottom fixed. Fractures were randomly positioned with 3 initial orientations: 0°, 60°, and 120°. The initial length of all cracks was set at 30 cm. Figure 4 illustrates one of these randomly generated initial configurations. Because of the loading conditions we would expect mode I failure, which is what we see in Fig. 4. The solid line lines in the bottom right panel represents the path to failure which is accurately predicted by the Random Forest model. The dashed indicates crack growth and coalescence in the HOSS simulations which are not captured in the Random Forest model. Crack propagation is simulated until complete material failure occurs. Due to its prior success and the small number of datasets (20 simulations), RFs and DTs have been employed to predict the time to failure. In addition to time to failure, the failure path is predicted based on cracks most likely to propagate and coalesce. Figure 5 shows the predicted times to failure for HOSS, DTs and RFs. The number of fractures oriented for mode I failure, and the maximum distance between neighboring fractures were the features extracted from each simulation. The importance of these features was verified with feature selection algorithms and resulted in the highest estimators’ accuracy. The agreement between the various predictive models (ML and analytical) and the high fidelity (i.e., HOSS) results are quantified in terms of an R^2^ value. The RF method performs worse than the DT method due to the small amount of training data used. For both of these models, the R^2^ is expected to increase with more training data until the point where additional data would only result in overfitting and yield diminishing returns. Representing spatial domains with RF and DTs is a new approach that has been highly successful for this crack propagation dataset. These results take the first steps in proving our third hypothesis that dominant emergent phenomena related to fracture interaction and coalescence can be predicted using ML methods.

## Discussion

The proposed methodology integrates computational physics, machine learning and graph theory to make a paradigm shift from computationally intensive high-fidelity models to coarse-scale graphs without loss of critical structural information. The underlying structure of fracture networks is critical to the dominant physics governing the system and the graph-based approaches offer great promise in collapsing often neglected, yet key microstructural information, into a compact representation. This key concept allows for a wider range of scales previously considered impossible to be captured at once. We demonstrated the utility of the method on two important geo-material problems, flow through fractured media and fracture propagation.

For the flow problem, the method allows us to easily identify primary flow paths without running flow and transport simulations. For the dynamic fracture problem, we refer to the path to material failure through the growth and coalescence of cracks that exhibit specific initial characteristics under certain loading conditions.

**Figure 6:**
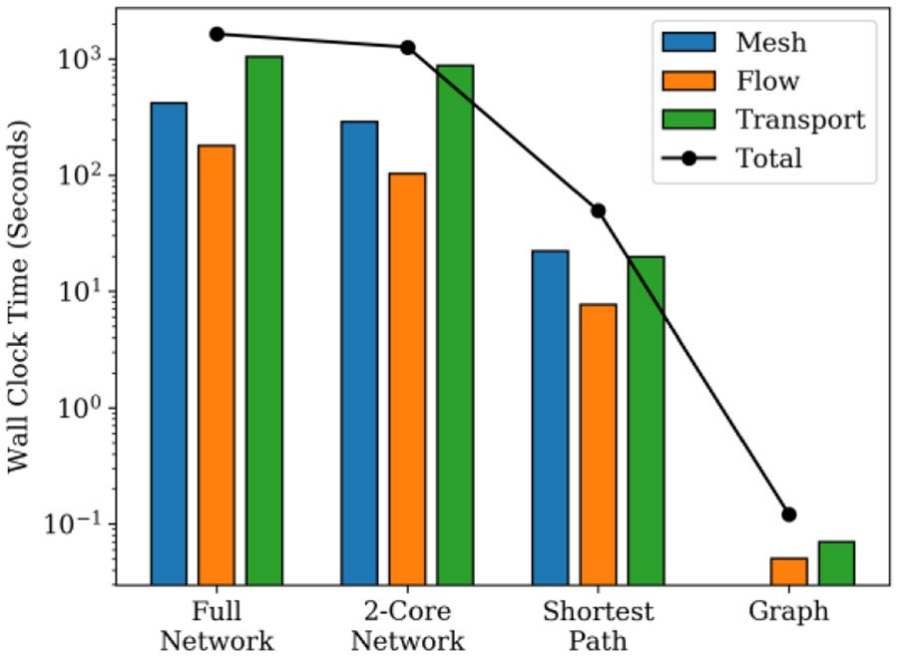
CPU times for dfnWorks suite - meshing, flow and transport solvers for the different fracture networks obtained by pruning vs. the full DFN.

We pruned discrete fracture networks based on two separate topological considerations, the aggressive shortest path and the conservative 2-core, which yielded vastly different results, each suitable for different scenarios. The results indicate that the shortest path is sufficient to accurately predict first arrival times with computations performed on only 10% of the original network. Since our solvers scale as O(N^2^), this results in four orders of magnitude computational savings (Fig. 6). This result is significant in the nuclear nonproliferation scenario of detection of chemical signatures following underground explosions. The objective is to detect trace particles of Xenon gas once it has migrated upwards to the atmosphere. The highest-level decision is when and where one might expect to fly over with detectors to optimize air sample collection, and the shortest path provides sufficient information to inform that decision. First arrival times are also crucial for answering questions about contamination of groundwater resources as a result of underground nuclear waste repositories or CO_2_ sequestration initiatives. In order to determine policy in a risk-informed manner, it is beneficial to consider a wide range of topological, geophysical and geometric configurations to bound the overall system-level uncertainties. Our graph/ML algorithms help us explore this vast uncertainty space in an efficient manner. On the other hand, the more conservative 2-core pruning algorithm results in a close match with the full network for the entire breakthrough curve except at very late times. For applications of oil and gas or hydrothermal extraction, the entire production curve is of relevance and the 2-core approximation can quickly provide insight into optimal locations for drilling production wells to maximize extraction by performing several thousand simulations with varying target locations.

Fractured systems have inherent topology uncertainty that dominants system behavior. Traditional UQ methodologies typically vary the parameters for a given (fixed) fracture network to determine the variations in the QOI, but this approach fails to account for the dominant topological uncertainty. Hence, comprehensive uncertainty quantification for these systems require 1000s of runs in a Monte Carlo framework varying the topology as well as geometry and physical properties. This step requires generating a different mesh in each instance, which even after the primary flow paths and reduced network have been identified, is a significant computational endeavor. This burden is the main motivation to turn to graph-based reduced order models as an appealing mesh-free alternative. Here the fracture characteristics are collapsed into edges with nodes representing fracture intersections. This assumption however comes with a cost in terms of reduced accuracy, or a systematic deviation from the high-fidelity solver operating on *F*.

The BTCs are nearly identical for small homogeneous systems, but for larger networks, we observe systematic deviations that increase with the size of the network^18^. Our UQ approach not only accounts for this systematic deviation through a calibration parameter, but also formulates a discrepancy term to account for the incomplete physical representation (see equation 1 in the Methods section). In the case presented here, possible systematic discrepancies arise due to reasons including but not limited to pruning the domain and simplifying the transport physics on the graph. Given 1000s of BTC_G and applying the calibration and discrepancy adjustment to each one results in 1000s of predictions of BTC_F with uncertainty without the need to compute 1000s of BTC_Fs. Estimates based on BTC_G (Fig. 3c) closely track those based on BTC_F, demonstrating that we can recover uncertainty bounds on the system accurately without the need to compute 1000s of expensive BTC_Fs making UQ feasible for more complex problems than previously possible. In the UQ method we acknowledge the simplified physics on the graph-based models in the tradeoff between accuracy and efficiency and account for it through modeling the calibration parameters and model form discrepancy. Our method is also agnostic to the size of the fracture network except in the small training set requiring DFN-graph pairs to learn the calibration and discrepancy.

The final part of this research addresses the complexity of fracture networks and the consequent computational burden that is limiting for large length scale modeling tools through the development of a coupled ML and graphical modeling approach. The premise of domain reduction for the brittle material failure problem is the assumption that clearly defined paths to failure exist and can be predicted *a priori* based on cracks characteristics and the extent of their interactions. Our eventual goal is to demonstrate the methodology on a simple fracture propagation example recognizing that our algorithms need to be further refined for more complex scenarios. Due to the promising predictions for the time and location of material failure (Figs 4, 5), the corresponding choice of features directed us to the driving factors of fracture network growth. As expected in mode I failure, fractures that are perpendicularly oriented to the load tend to propagate faster than other orientations. Additionally, location and the distance between fracture tips play a vital role in inter-fracture connections. The amount of data has limited our analysis to relatively basic ML algorithms that have constrained predictive abilities. It has been shown that even with these under-informed approaches, significant trends in fracture network growth and the time to material failure can be found.

The insight on spatial and temporal material failure will lead to the building of larger predictive machines that can handle the wide range of conditions a material can be subject to. Our ongoing work utilizes a dataset that is multiple orders of magnitude larger. Initial fracture lengths, magnitude of axial load, fracture density, and geometries are all routes that are being analyzed in more depth. Despite the small amount of training data and relatively basic ML algorithms considered in this study, we see that significant trends in fracture network growth and the time to material failure can be found. The insight on spatial and temporal material failure will lead to the building of larger predictive machines that can handle the wide range of conditions a material might be subjected to.

## Methods

The overall objective of this work is to develop reduced order hybird graph-based / machine learning representations of high fidelity simulators to answer key science questions regarding the physics in fracture networks. The flow and transport simulations on the fracture networks are run using the dfnWorks suite, developed at Los Alamos by this research team. dfnWorks combines the feature rejection algorithm for meshing (FRAM)^23^ to create conforming Delaunay triangulations of the DFN using the LaGriT meshing toolbox^7^, the parallelized subsurface flow code PFLOTRAN^24^, and a particle tracking method. dfnWorks has been used in a variety of studies including hydraulic fracturing^25,26^ and parameter assessment for subsurface flow and transport in large fracture networks^27,28^.

We use dfnWorks to generate a discrete fracture network representation of fractured systems and simulate flow and transport therein. The next step is to construct a graph representation *G* of the DFN *F* based on the topology alone. Fractures are mapped to nodes in the graph and edges exist between nodes if the two fractures that they represent intersect one another. This mapping is an isomorphism that allows us to switch between *F* and *G* uniquely. Source and target nodes are attached to nodes whose corresponding fractures intersect the inflow and outflow boundaries respectively^7^. We then find the shortest path and the 2-core representations on this directed graph using standard algorithms in NetworkX^29^. The reduced graph representations are mapped back to fracture networks and we use dfnWorks to generate the mesh, and run flow and transport solvers to produce breakthrough curves on the pruned network. Since the numerical solvers within dfnWorks scale roughly as *O*(N^2^), where N is the number of mesh elements, the computational speedup from replacing F with F′ is significant, as seen in Fig. 6.

The transport algorithm on the graph solves Laplace’s equation for flow and transport is performed using particle tracking on a graph representation of fracture networks^18,30^. In this representation, nodes are midpoints of fracture intersections and edges represent flow pathways on fractures between intersections. The graph method which solves Laplace’s equation is derived from balance of mass on graph nodes along with an equivalent Darcy model where the mass flux is proportional to the pressure gradient across two graph nodes. While this methodology is 4 orders of magnitude faster than the above-mentioned method of reverting back to the fracture network and applying the dfnWorks suite to obtain the BTCs, the approximations made in the reduced order graph model result in systematic deviations.

We apply our Bayesian UQ methodology to correct for the observed systematic deviations as follows. The function f(BTC_G, θ) may be a simple time scaling of BTC_G where θ captures the magnitude of the shift. The discrepancy is often a smooth function in time and modeled with a Gaussain process, which is a distribution on smooth functions^31,32^. The relationship is as follows:

$${\rm{B}}{\rm{T}}{\rm{C}}{\rm{\_}}{\rm{F}}({\rm{t}})={\rm{B}}{\rm{T}}{\rm{C}}{\rm{\_}}{\rm{G}}({\rm{t}}+\theta )+\delta ({\rm{t}})\,$$

where the θ are calibration parameters and δ is the discrepancy. Here, the relationship between BTC_F and BTC_G is decomposed into two components. The first is a calibration component where we learn the value of θ that minimizes the difference between BTC_F(t) and BTC_G(t + θ) across all pairs of BTCs with uncertainty. The second component captures the unexplained difference between BTC_F(t) and calibrated BTC_G(t + θ) referred to as discrepancy. The discrepancy is often a smooth function in time and modeled with a Gaussian process, which is a distribution on smooth functions^31,32^. We start by learning the relationship between BTC_F and BTC_G from a small number of distinct simulations of transport on F generated using known fracture statistics and the corresponding G. Thus, the computationally expensive BTC_F is only computed a small number of times. We then quantify the uncertainties by performing transport calculations on 1000 s of computationally inexpensive G derived from the corresponding F’s. We validate our UQ approach both at the individual BTC_F scale and the system scale via posterior predictive checks^33^. Given a BTC_G, we modify it via calibration and a discrepancy adjustment, resulting in a prediction for BTC_F with uncertainty. In the training and validation phase, we compare the known BTC_F to the predictions to ensure that the actual BTC_F does lie within the uncertainty bands predicted for BTC_G.

The fracture propagation simulator HOSS^6^ is a discrete-element finite element analysis tool that can account for the complexity of a fracture network’s growth over periods of time. HOSS can resolve individual micro-cracks unlike the macro-scale continuum models, and can also account for interactions between micro-cracks in addition to coalescence and growth. As previously mentioned, this software can result in billions of unknowns for a relatively small system (10^6^ cracks) resulting in a computationally infeasible problem on the macro-continuum scale.

For the dynamic fracture propagation scenario, the first step is the selection of key features including initial lengths, orientations, loading conditions, and fracture propagation rates obtained from performing HOSS simulations. These features are imported into a graphical model where an individual fracture and its properties (orientation, geometry, etc.) are represented by a node and the features of that node. The subsequent step is to allocate a certain percentage of the data for training the ML model and the remaining data for validation of that model.

The ML algorithms employed for 20 micro-crack fracture networks are Decision Trees (DT) and Random Forests (RF). These algorithms provide great insight on feature importance within the model and data for smaller datasets such as in this study, which used 20 simulations total. DTs cycle through all the feature vectors (properties extracted from a dataset or model) and labels (features that are being predicted), then finds the best feature to split the data on. Usually, this split point is where the standard deviation between the two resulting groups are minimized. RFs are considered ensemble models since they are composed of many smaller models, while DTs consist of a single model. RFs cycle and split datasets in a very similar fashion to DT. The main difference, instead of cycling through the entire dataset with one DT, the dataset is split up and trained on separate DTs, hence the name RFs. The final prediction for RFs is a weighted average of all the separately trained models. A depth of 3 was used for both RFs and DTs.

These predictive tools are compared with HOSS results. The DT and RF were trained and tested with Leave-One-Out Cross-Validation using N-1 data points for training while testing on the one held out data point. Initial configuration information (distance between fracture tips, orientation, length, etc.) provides the ML model with training data and corresponding labels are the times when the material fails.

### Data availability

The datasets generated and analyzed during the current study are available from the corresponding author upon request.
